# Pharmic Activation of PKG2 Alleviates Diabetes-Induced Osteoblast Dysfunction by Suppressing PLC*β*1-Ca^2+^-Mediated Endoplasmic Reticulum Stress

**DOI:** 10.1155/2021/5552530

**Published:** 2021-06-16

**Authors:** Tingting Jia, Ya-nan Wang, Yao Feng, Chenchen Wang, Dongjiao Zhang, Xin Xu

**Affiliations:** ^1^Department of Implantology, School and Hospital of Stomatology, Cheeloo College of Medicine, Shandong University, Jinan, Shandong 250012, China; ^2^Shandong Key Laboratory of Oral Tissue Regeneration, Jinan, Shandong 250012, China; ^3^Shandong Engineering Laboratory for Dental Materials and Oral Tissue Regeneration, Jinan, Shandong 250012, China; ^4^Department of Oral Medicine, Qilu Hospital of Shandong University, Jinan, Shandong 250012, China

## Abstract

As reported in our previous study, cinaciguat can improve implant osseointegration in type 2 diabetes mellitus (T2DM) rats by reactivating type 2 cGMP-dependent protein kinase (PKG2), but the downstream mechanisms remain unclear. In the present study, we investigated the favorable effect of cinaciguat on primary rat osteoblast, which was cultivated on titanium disc under vitro T2DM conditions (25 mM glucose and 200 *μ*M palmitate), and clarified the therapeutic mechanism by proteomic analysis. The results demonstrated that T2DM medium caused significant downregulation of PKG2 and induced obvious osteoblast dysfunction. And overexpression of PKG2 by lentivirus and cinaciguat could promote cell proliferation, adhesion, and differentiation, leading to decreased osteoblasts injury. Besides, proteomic analysis revealed the interaction between PKG2 and phospholipase C*β*1 (PLC*β*1) in the cinaciguat addition group, and we further verified that upregulated PKG2 by cinaciguat could inhibit the activation of PLC*β*1, then relieve intracellular calcium overload, and suppress endoplasmic reticulum (ER) stress to ameliorate osteoblast functions under T2DM condition. Collectively, these findings provided the first detailed mechanisms responsible for cinaciguat provided a favorable effect on promoting osseointegration in T2DM and demonstrated a new insight that diabetes mellitus-induced the aberrations in PKG2-PLC*β*1-Ca^2+^-ER stress pathway was one underlying mechanism for poor osseointegration.

## 1. Introduction

Type 2 diabetes mellitus (T2DM) is a common metabolic disease characterized by hyperglycemia and dyslipidemia [1], which is associated with osseous complications, such as osteoporosis, fractures, or osteoarthrosis [2–4]. It also acts as a risk factor for the process of osseointegration, even lead to titanium implant loss or lost due to impaired wound healing [5], and subsequent bone resorption. Multiple studies have indicated that patients with diabetes have an increased risk for implantation failure than that of nondiabetics [6]. Numerous theories have been proposed for the etiology of diabetes-mediated poor implant osteointegration, such as dysfunction of mitochondria [7] and accumulation of reactive oxygen species [8] in osteoblasts. Meanwhile, our previous study has demonstrated that the activity of type 2 cGMP-dependent protein kinase (PKG2) was decreased in the peri-implant bone area, and cinaciguat treatment provided a favorable effect on promoting osseointegration in T2DM rats [9]. However, detailed cellular mechanisms of these findings are still unclear and need further investigations.

Cyclic GMP (cGMP) plays a critical role in maintaining glucose metabolism and bone homeostasis, which is produced by soluble guanylyl cyclases (sGCs) and hydrolyzed by phosphodiesterase-5 (PDE5) [10]. Then, cGMP can be regarded as secondary messenger to activate two types of protein kinases (PKG1 and PKG2) [11]. The expression of PKG1 is limited in the vasculature, and PKG2 is mainly expressed in specific cell types, such as bone, intestine, brain, and kidney [12]. Increasing literatures have shown that PKG2 is a key modulator in skeletal homeostasis and bone remodeling. The PKG2-deficient mice developed dwarfism that was caused by interfered with endochondral ossification [13]. And treatment with PKG2-generating agents could restore osteoblast function and bone formation in diabetes [14]. In recent years, the impaired cGMP-PKG2 pathway in various tissues has been stated to be associated with the risk of diabetes and diabetic complications, for instance, cardiomyopathy [15] and nephropathy [16]. Our research group also demonstrated that low expression of PKG2 and high expression of PDE5 appeared in the peri-implant bone in diabetic rats [9]. Therefore, drugs are demanded to reactivate the interfered cGMP/PKG2 pathway to exert its protective effect in T2DM conditions.

Cinaciguat (BAY58-2667) was selected from 800 analogues as the first NO-independent activator of sGC in 2002 [17], which could activate sGC in the low nanomolar range, thereby high-efficiently generate cGMP-producing activity and augment PKG2. Several studies have reported its beneficial effects in heart failure [18], pulmonary hypertension [19], and erectile dysfunction [20]. Especially, cinaciguat was considered to be a novel treatment target for osteoporosis, which enhances osteocyte survival in OVX mice [21]. Recent studies have shown that cinaciguat could play protective action in hyperglycemic environments to improve cardiac dysfunction and prevent bone loss of diabetic animals. Moreover, we concluded that long-term use of cinaciguat could improve poor implant osteointegration in T2DM rats. This favorable effect of cinaciguat was delivered by normalization of PKG2 expression, but little information is available about the downstream mechanisms.

With the development of high-throughput proteomic sequencing, much progress has been made to elucidate the underlying proteins involved in diabetes development. In the current study, we examined the role of reactivation PKG2 on osteoblasts, which were cultured on titanium sheets in vitro T2DM models, and hypothesized that osteoblasts would exhibit an ameliorative osteogenic ability when cultured with cinaciguat. Furthermore, isobaric tag for relative and absolute quantitation (iTRAQ) of osteoblasts in T2DM environment with and without cinaciguat treatment was conducted to clarify the underlying molecular mechanism, thus, we found the reduced expression of phospholipase C*β*1 (PLC*β*1) may play a vital role in this process. We supposed that the excessive activation of PLC*β*1 under diabetic microenvironment caused stimulation of phosphatidylinositol 4,5-bisphosphate (PIP2) hydrolysis and inositol 1,4,5-trisphosphate (IP3) generation, inducing IP3-dependent calcium overload and endoplasmic reticulum (ER) stress, eventually lead to poor osteointegration. Fortunately, reactivation PKG2 by cinaciguat could rescue this adverse process. Overall, our study elaborated a new mechanism of PKG2-PLC*β*1-Ca^2+^-ER stress axis, which could be a potential molecular target for further intensive exploration regarding the effect of T2DM on implant osteointegration.

## 2. Materials and Methods

### 2.1. Primary Rat Osteoblast Culture and Treatment

Neonatal Sprague-Dawley rats (<24 h old) were selected for osteoblast isolation, which were approved by the Animal Ethics Committee of the School of Stomatology, Shandong University. Osteoblasts were primarily obtained from the calvaria of rat by tissue block method and purified using repetitive adhesion. Cells were cultured in Dulbecco's modified Eagle's medium (DMEM, BI, USA), supplemented with 10% fetal bovine serum (FBS, BI, USA) and 1% penicillin/streptomycin, at 37°C under a humidified atmosphere of 5% CO_2_. The morphology and growth of cells were observed by an inverted microscope. Also, the isolated and purified osteoblasts were identified by alkaline phosphatase staining and alizarin red staining. All osteoblasts at cell passages 3-5 were seeded on Titanium (Ti) discs (Grade 1; Baoti, Shanxi, China) in different plates.

To detect the role of PKG2 activation in T2DM-induced osteoblast dysfunction, cells were cultured in different groups, including normal medium (5.5 mM glucose), control medium (adding 20 mM mannitol to the 5 mM glucose medium and palmitate vehicle), T2DM medium (25 mM glucose and 200*μ*M palmitate to mimic diabetic conditions) [22], T2DM medium with cinaciguat (100 nM, MedChemExpress, USA), or T2DM medium with m-3M3FBS (0.5 *μ*M, Santa Cruz, USA), U73122 (2 *μ*M, MedChemExpress, USA), 4-PBA (200 *μ*M, MedChemExpress, USA), Tunicamycin (0.2 *μ*g/ml, MedChemExpress, USA).

### 2.2. Cell Proliferation Assessments

EDU assay kit (RiboBio, China) was used to test the proliferation of osteoblasts based on the manufacturer's instructions. Cells were seeded in Ti disc (diameter 10 mm) placed in a 48-well plate (10^4^ cells/well). EDU detection was monitored 3 days after cells seeding. Different culture medium was replaced by 50 *μ*mol/L EDU labelling medium and incubated for an additional 2 h at 37°C. Then, cells were fixed with paraformaldehyde, enhanced permeability with 0.5% TritonX-100, and stained with Apollo®567 solution and Hoechst 33342 solution. Finally, the osteoblasts were observed by fluorescence microscope (Leica, Germany). The percentage of EDU-positive cells (red fluorescence) among the total cells (blue fluorescence) was calculated by ImageJ software.

### 2.3. Cell Apoptosis Assessments

The apoptosis of different group cells was detected by flow cytometry kit (Liankebio, China). Briefly, osteoblasts were harvested and centrifuged for 3 min at 1000 rpm, then resuspended in 1x Annexin V binding solution containing 5 *μ*l Annexin V-FITC and 10 *μ*l PI for 10 min at room temperature in dark. The apoptosis rate was acquired by flow cytometer (BD Accuri TM, USA), and data were analyzed with BD Accuri TMC6 Plus Software.

### 2.4. Cell Adhesion Assessments

Rhodamine-phalloidin staining was employed to observe the osteoblast morphology, which was cultured on Ti discs with different treatments. After fixed with 4% formaldehyde, cells were washed with PBS and 0.5% Triton X-100, then stained with Rhodamine-phalloidin and Dapi for observation by fluorescence microscope (Leica, Germany).

### 2.5. Osteogenic Differentiation Assessments

Alkaline phosphatase (ALP) activity assay, ALP staining, and alizarin red staining were used to measure the cell osteogenetic ability under different conditions. At day 7 after induction, the ALP activity assay was performed following the instructions of the ALP activity assay kit (Nanjing Jiancheng, China), and the absorbance at 520 nm was measured by microplate spectrophotometer. For staining, cells were washed thrice with phosphate-buffered saline (PBS) and fixed in 4% paraformaldehyde, then stained with NBT/BCIP staining kit (Beyotime, China) or Alizarin Red (Solarbio, China), and finally washed twice with ddH_2_O.

### 2.6. Cell Immunostaining Staining

Immunofluorescence staining was used to examine the expression levels and nuclear-cytoplasm distribution of PKG2 or PDE5. The cells were seeded on Ti disc in 12-well plate and treated with different stimuli. Three days after treatment, osteoblasts were washed with PBS, fixed with paraformaldehyde, blocked with 1% BSA, and incubated with primary antibodies to PKG2 (Santa Cruz, sc-393126, 1 : 200, USA) and PDE5 (Abcam, ab14672,1 : 500, UK) overnight at 4°C in sequence. Subsequently, the cells were incubated with fluorescent secondary antibodies (goat anti-rabbit IgG, Abcam, ab150084, 1 : 400, UK; goat anti-mouse IgG, Abcam, ab150117, 1 : 400, UK) in the dark, and the nucleus was stained by DAPI (Sigma, USA). The positive cells were visualized by fluorescence microscopy (Leica, Germany), and the fluorescent quantitative analysis was conducted by Image J software.

### 2.7. Protein Isolation and Western Blot

Proteins were extracted from osteoblasts by RIPA lysis buffer (Solarbio, China) on ice, and the supernatant lysate was collected after centrifugation at 12,000 rpm for 15 min. Then, the concentration was measured using BCA Protein Assay Kit (Solarbio, China). After protein denaturation for 5 min at 100°C, the proteins were separated on SDS-polyacrylamide gels and transferred to polyvinylidene difluoride (PVDF) membranes by a wet transfer apparatus. Bands were blocked in 5% dried milk for 1 h and incubated overnight at 4°C with first antibodies against COLI (Cell Signaling Technology, CST, 84336, 1 : 1000, USA), ALP (Abcam, ab108337, 1 : 1000, UK), RUNX2 (CST, 8486, 1 : 1000, USA), PKG2 (Santa Cruz, sc-393126,1 : 500,USA), PDE5 (Abcam, ab14672,1 : 1000, UK), PLC*β*1 (Abcam, ab182368, 1 : 1000, UK), IP3R (Santa Cruz, sc-377518,1 : 500,USA), PERK (Bioss, bs-2469R, 1 : 500, China), phospho-PERK (Bioss, bs-3330R, 1 : 500, China), GRP78 (Bioss, bs-1219R, 1 : 500, China), ATF4 (Bioss, bs-1531R, 1 : 500, China), CHOP (CST, 2895, 1 : 1000, USA), *β*-actin (Abways, AB0035, 1 : 5000 China), and GAPDH (Proteintech, 10494-1-AP, 1 : 5000, China). The next day, the membranes were washed and incubated with anti-rabbit or anti-mouse HRP-conjugated secondary antibodies. The protein bands were visualized under Amersham Imager 600 (Millipore, USA), and intensities were calculated by ImageJ software.

### 2.8. Transmission Electron Microscopy (TEM) Investigations

TEM investigations were conducted to confirm the dilation of endoplasmic reticulum. Osteoblasts were harvested and fixed in 3% glutaraldehyde at room temperature. After washed by 0.1 M phosphate buffer (PBS), cells were fixed again by 1% osmic acid, then, the samples were subjected to gradient ethanol dehydration, acetone penetration, embedding, and polymerized in a drying oven for 12 h at 37°C, 12 h at 45°C, and 48 h at 60°C. Subsequently, ultrathin sections (50-70 nm) were sliced and stained with 2% uranyl acetate and lead citrate. Photos were acquired using transmission electron microscope (FEI F200C, USA).

### 2.9. siRNA Knockdown and Lentivirus-Mediated PKG2 Overexpression of Osteoblasts

Targeted small interfering RNA (siRNA) was designed to knock down PKG2 gene expression by Genepharma (Shanghai, China). Osteoblasts were transfected using Micropoly-transfecter (Jiangsu, China), and knockdown efficiency was detected by total RNAs and proteins, which were extracted at 48 h or 72 h after transfection.

For overexpression, lentivirus packaging osteoblasts were transfected with LV-Prkg2 and LV-Con purchased from Genechem (Shanghai, China). After infection, cells were stably selected with puromycin (Solarbio, China).

### 2.10. Proteomic Analysis

Osteoblasts from T2DM medium and T2DM medium with cinaciguat were prepared as samples for proteomic analysis with three biological replicates for each group. Six groups of protein were separated, and the concentrations were determined with the BCA method, then SDS-polyacrylamide gels electrophoresis was performed for protein quantification. After proteolysis with Filter-Aided Sample Preparation (FASP), the protein samples were separately labeled by TMT kit (Thermo Fisher Scientific, USA) according the manufacturer's instructions. Subsequently, the labeled peptides of each group were mixed and fractionated by 1260 infinity II high-performance liquid chromatography (Agilent, Germany), and mass spectrometry (MS) analysis was performed.

For bioinformatic analysis, the GO and KEGG pathway enrichment analyses were performed to identify candidate biomarkers by the Fisher's Exact Test. For cluster analysis, the quantitative information of the target protein was normalized firstly, then, we used Matplotlib software to classify the two dimensions of sample and protein expression at the same time and generated hierarchical clustering heat map. The Search Tool for the Retrieval of InteractingGenes/Proteins (STRING) database and Cytoscape software were used to generate a protein-protein interaction (PPI) network.

### 2.11. Determination of Intracellular Ca^2+^ Concentration

The concentration of intracellular Ca^2+^ was measured in osteoblasts using a fluorescent Ca^2+^ indicator Calbryte™ 520 AM (AAT Bioquest, USA). After washing by Hanks and Hepes Buffer (HHBS), 200 *μ*L of 2 *μ*M Calbryte™ 520 AM was added to the wells and incubated for 45 min at 37°C and then incubate the plate at room temperature for another 15 min. Replace the dye working solution with HHBS, and Images were acquired in the FITC channel.

### 2.12. Statistical Analysis

All data were expressed as mean ± standard deviations (SD) at least three independent experiments. Statistically significant differences (*p* value of less than 0.05) were analyzed using two-tailed Student's *t*-test and one-way analysis of variance (ANOVA) with SPSS 16.0 software.

## 3. Results

### 3.1. Impaired Proliferation, Adhesion, and Differentiation Capability of Osteoblast in T2DM Medium

**Figure 1:**
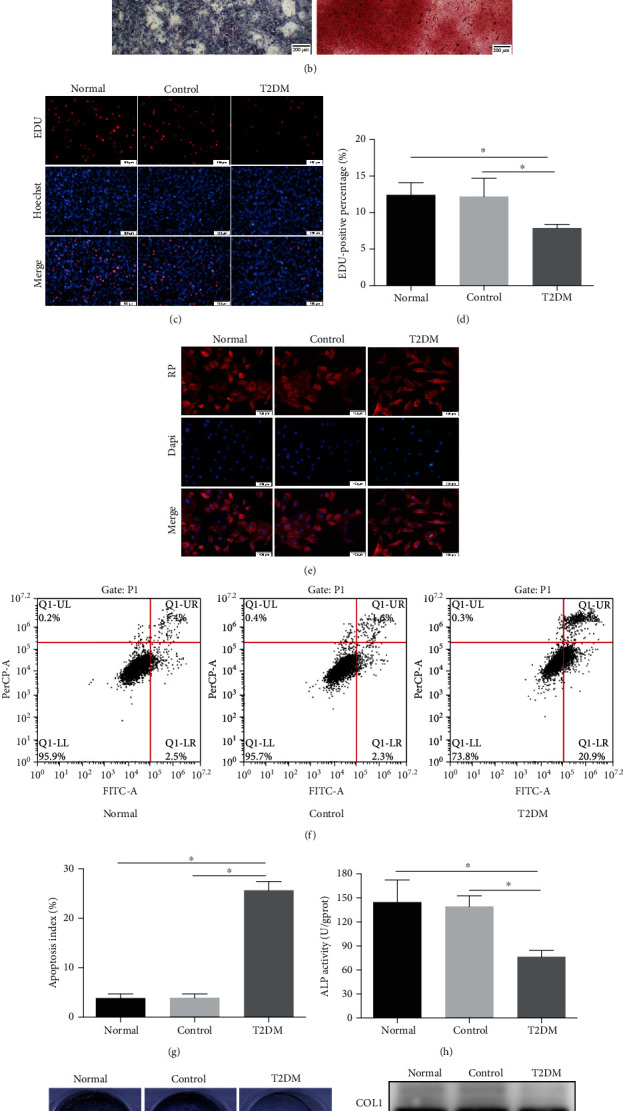
Impaired proliferation, adhesion, and differentiation capability of osteoblast in T2DM medium. (a) The morphology of osteoblast, left: primary culture, right: passages P3. Scale bar = 200 *μ*m. (b) The identification of osteoblast, left: ALP staining, right: Alizarin red Staining. Scale bar = 200 *μ*m. (c) EDU staining was performed to test the osteoblasts undergoing DNA replication. Red, EDU positive nucleus. Blue, all nucleus. Scale bar = 100 *μ*m. (d) The quantification analysis of EDU-positive cells. (e) Cytoskeleton (red) was stained by rhodamine-phalloidin, and nucleus (blue) was stained by DAPI. Scale bars = 100 *μ*m. (f) Flow cytometer analysis was conducted to detect cells apoptosis. (g) The quantification analysis of apoptosis rate. (h) ALP activity assay after 7 days induction. (i) ALP staining (upper) and Alizarin red staining (lower) after 7 days or 28 days induction. (j) The protein levels of COL1, ALP, and RUNX2 were detected by western blot after 7 days induction. Data were presented as means ± SD from at least three independent experiments. ∗: *p* < 0.05. RP: rhodamine-phalloidin.

In this study, we first identified the purity of the rat primary osteoblast. After three sample purifications, the isolated cells exhibited typical morphology of osteoblasts with good proliferation ability (Figure 1(a)). ALP staining showed positive results, and alizarin red staining presented calcium nodules with different sizes (Figure 1(b)), which indicated the cells possessed osteogenetic property.

Meanwhile, we mimicked diabetic conditions in vitro by using 25 mM glucose and 200 *μ*M palmitate, as previously reported [22]. To investigate whether T2DM medium had a profound effect on many aspects of osteoblasts, we explored its proliferation, adhesion, and differentiation capability. EDU test displayed that T2DM-treated osteoblasts had reduced proliferation ability relative to normal group, as revealed by less EDU-positive cells (Figures 1(c) and 1(d)). T2DM medium also induced more cell apoptosis, as quantified by evaluating Annexin V-FITC and PI staining (Figures 1(f) and 1(g)). Cell adhesion was directly qualitative analysis by Rhodamine-phalloidin staining 48 h after stimulation on Ti surfaces. We could find that osteoblasts in the normal and control group appeared more stretched and larger than cells in the T2DM group (Figure 1(e)). After treating with osteogenic induction medium for 7 days and 28 days, the ALP activity and Alizarin red staining were obviously reduced upon T2DM medium addition (Figures 1(h) and 1(i)). Consistently, T2DM medium led to lower expression of osteogenesis-related protein (including COL1, ALP, and RUNX2) detected by western blotting compared with the normal group (Figure 1(j)). And the differences were not from elevated osmolarity and vehicle, because the control group neither affected cell viability nor cell differentiation.

### 3.2. Downregulation of PKG2 Expression in Osteoblast Culturing with T2DM Medium

**Figure 2:**
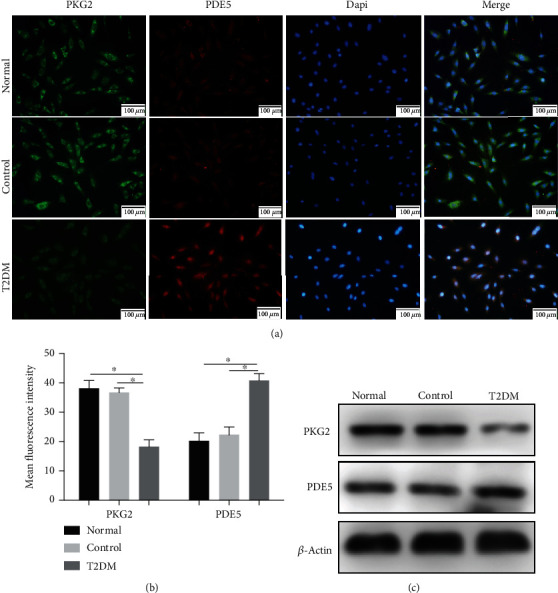
Downregulation of PKG2 expression in osteoblast culturing with T2DM medium. (a) Immunofluorescent double staining was performed to examine the expression of PKG2 and PDE5 after 3 days of T2DM stimulation. Scale bars = 100 *μ*m. (b) Quantitative analysis of mean fluorescence intensity of PKG2 and PDE5. (c) The protein levels of PKG2 and PDE5 were detected by western blot after 3 days of T2DM stimulation. Data were presented as means ± SD from at least three independent experiments. ∗: *p* < 0.05.

Existing evidences have demonstrated that the cGMP/PKG2 pathway could be deteriorated under diabetic conditions, and this phenomenon was also proved by Figure 2. Western blot showed that the downregulated expression of PKG2 and upregulated expression of PDE5 were observed in the T2DM group. Immunofluorescence displayed a further verification to western blot, primarily manifested as a stronger red fluorescence (PDE5) and weaker green fluorescence (PKG2) under T2DM condition. The above results demonstrated that PKG2 was suppressed by diabetes.

### 3.3. Overexpression of PKG2 Relieved Osteoblast Dysfunction under T2DM Condition

**Figure 3:**
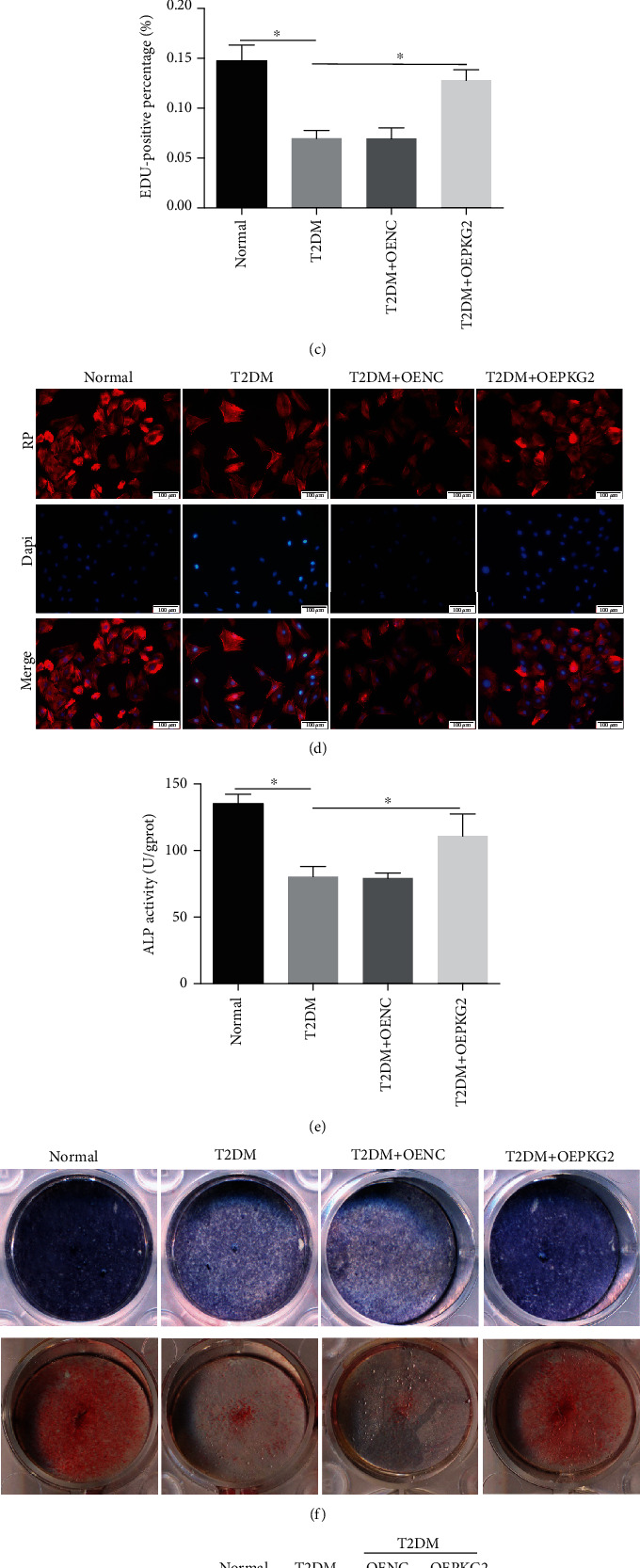
Overexpression of PKG2 relieved osteoblast dysfunction under T2DM condition. (a) The overexpression efficiency of PKG2 was verified by western blot. (b) EDU staining was performed to test the osteoblasts undergoing DNA replication. Red, EDU positive nucleus. Blue, all nucleus. Scale bar = 100 *μ*m. (c) The quantification analysis of EDU-positive cells. (d) Cytoskeleton (red) was stained by rhodamine-phalloidin, and nucleus (blue) was stained by DAPI. Scale bars = 100 *μ*m. (e) ALP activity assay after 7 days induction. (f) ALP staining (upper) and Alizarin red staining (lower) after 7 days or 28 days induction. (g) The protein levels of COL1, ALP, and RUNX2 were detected by western blot after 7 days induction. Data were presented as means ± SD from at least three independent experiments. ∗: *p* < 0.05. OENC: overexpression negative control; OEPKG2: overexpression of PKG2; RP: rhodamine-phalloidin.

To determine the effects of PKG2 on diabetes-induced osteoblast dysfunction, lentiviral vector was used to overexpress PKG2. The overexpression efficiency was examined by western blot (Figure 3(a)). Compared with the negative control (OENC) group, an obvious increase in cell proliferation was discovered in the T2DM-treated osteoblast after overexpression of PKG2 (Figures 3(b) and 3(c)). Also, the cytoskeleton of cells inclined to be extended and dense on Ti surfaces in the PKG2-higher expression group, while the T2DM and OENC groups acquired wizened and sparse cytoskeletal structure (Figure 3(d)). Furthermore, T2DM-induced decrease of differentiation capability in osteoblast was recovered by overexpression of PKG2. ALP activity and mineralization capacity (Figures 3(e) and 3(f)) were clearly increased in osteoblast, and the protein expression level of COL1, ALP, and RUNX2(Figure 3(g)) was upregulated because of transfected with lentiviruses PKG2. Taken together, overexpression of PKG2 in vitro reduced pathway dysfunction and promoted osteogenesis.

### 3.4. Cinaciguat Alleviates T2DM-Induced Osteogenesis Injury by Upregulating PKG2

**Figure 4:**
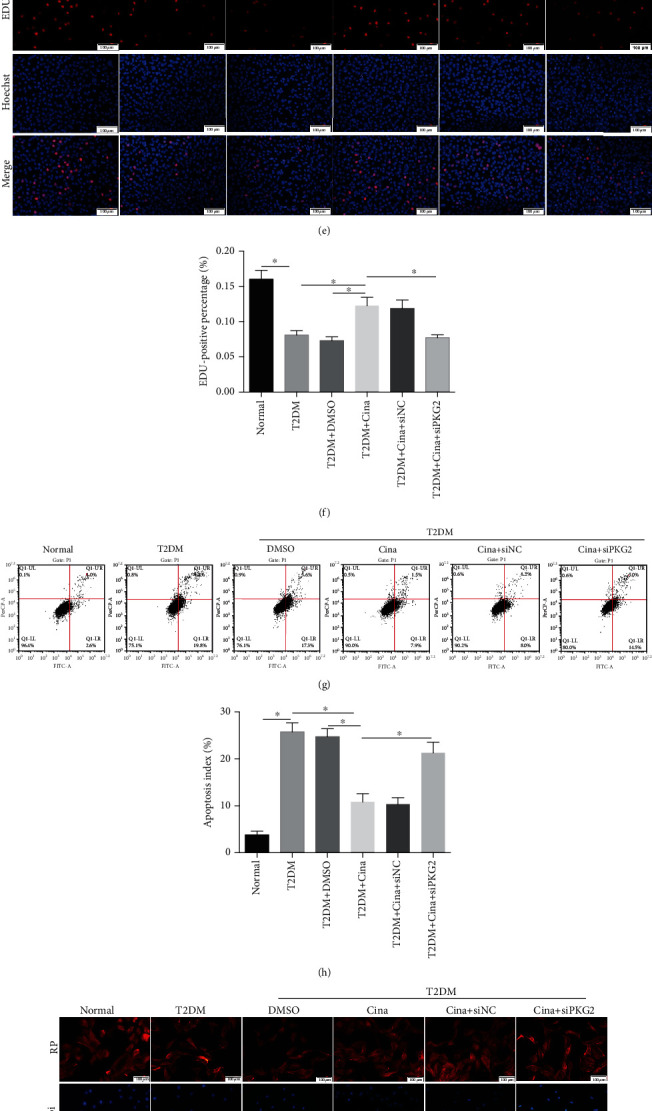
Cinaciguat alleviates T2DM-induced osteogenesis injury by upregulating PKG2. (a) The protein level of PKG2 was detected by western blot after 7 days of adding cinaciguat. (b) Immunofluorescent staining was performed to examine the expression of PKG2 after 3 days of adding cinaciguat. Scale bars = 100 *μ*m. (c) Quantitative analysis of mean fluorescence intensity of PKG2. (d) The knockdown efficiency of PKG2 was verified by western blot. (e) EDU staining was performed to test the osteoblasts undergoing DNA replication. Red, EDU positive nucleus. Blue, all nucleus. Scale bar = 100 *μ*m. (f) The quantification analysis of EDU-positive cells. (g) Flow cytometer analysis was conducted to detect cells apoptosis. (h) The quantification analysis of apoptosis rate. (i) Cytoskeleton (red) was stained by rhodamine-phalloidin, and nucleus (blue) was stained by DAPI. Scale bars = 100 *μ*m. (j) ALP activity assay after 7 days induction. (k) ALP staining after 7 days induction. (l) The protein levels of COL1, ALP, and RUNX2 were detected by western blot after 7 days induction. Data were presented as means ± SD from at least three independent experiments. ∗: *p* < 0.05. Cina: cinaciguat; RP: rhodamine-phalloidin.

Our previous study has confirmed that pharmacologic activation of the cGMP/PKG2 signaling by cinaciguat could improve implant osseointegration in T2DM rats [9], but its effects on osteoblast biological behavior on titanium surface in vitro remain unclear. First, western blot and immunofluorescence confirmed the disturbed pathway was ameliorated by cinaciguat, which mainly manifested as high-expression of PKG2 in T2DM-cinacguat group (Figures 4(a)–4(c)). Second, our work showed that PKG2 was necessary for the proproliferative and prosurvival effects of cinaciguat in osteoblasts under T2DM condition. Culturing osteoblasts in T2DM medium with cinaciguat recovered EDU incorporation (Figure 4(e)) and cytoskeletal structure (Figure 4(i)) and also reduced cell apoptosis to 10.8% (Figures 4(g) and 4(h)). The efficiency of knockdown PKG2 by siRNA was examined by western blot (Figure 4(d)). As expected, the favorable effects were faded down when knockdown of PKG2 with siRNA. Finally, adding cinaciguat to the T2DM medium obviously increased ALP production at 7 days (Figures 4(j) and 4(k)). Cinaciguat also induced an increase in expression of the osteoblastic differentiation marker proteins COL1, ALP, and RUNX2 (Figure 4(l)). Similarly, cinaciguat was ineffective to enhance osteogenesis in prkg2-deficient osteoblasts. These results demonstrated that cinaciguat could reverse the negative effects of diabetes on osteoblast in vitro, which was mediated by PKG2.

### 3.5. Quantitative Proteomic Analysis of Differentially-Expressed Proteins (DEPs) Regulated by Cinaciguat in Osteoblasts under T2DM Condition

**Figure 5:**
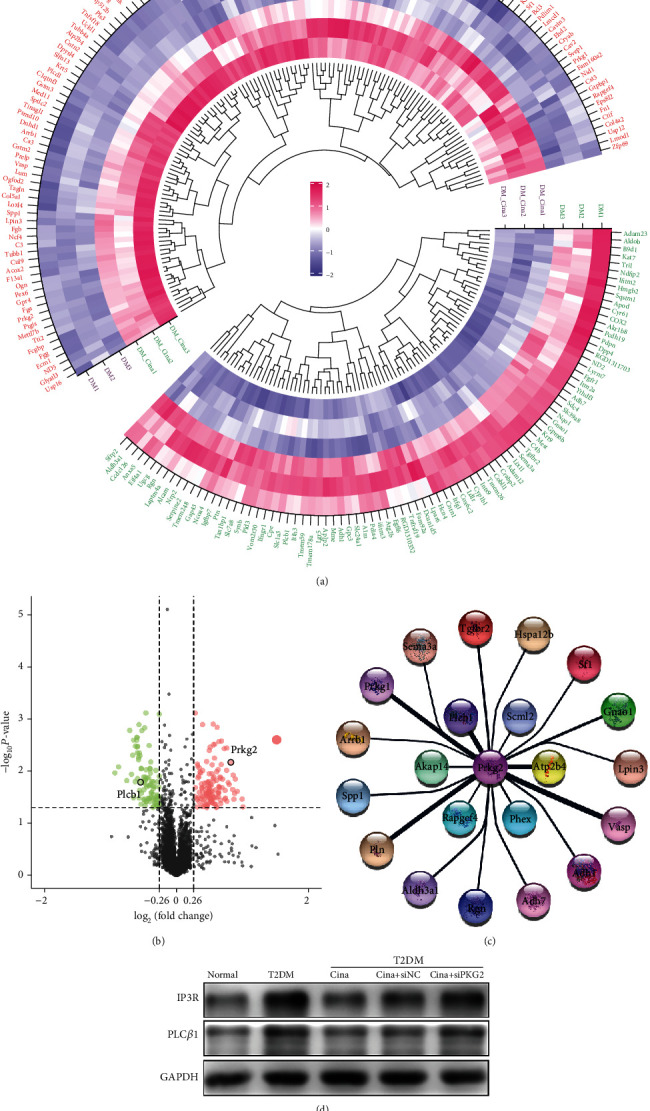
Quantitative proteomics analysis of differentially-expressed proteins regulated by cinaciguat in osteoblasts under T2DM condition. (a) Hierarchical clustering of the differentially expressed proteins. (b) Volcano plots of protein expression profiles. Red/green symbols classified the upregulated/downregulated proteins. (c) Protein-protein interaction was constructed by STRING platform, and the network was visualized in cytoscape. The thick lines represent strong association among proteins. (d) The protein levels of PLC*β*1 and IP3R were detected by western blotting to verify the protein mass spectrometry result. Cina: cinaciguat.

Further, we explored the potent molecular mechanisms that how upregulated PKG2 by cinaciguat affected the fate of osteoblasts in the T2DM medium. Protein components between the T2DM group and T2DM + cinaciguat group were compared by proteomic analysis. The identification of DEPs was based on two primary principles, *p* value < 0.05 and an absolute fold change ≥ 1.2. Thus, among a total of 6437 proteins quantified, 130 proteins were upregulated, and 91 proteins were downregulated after adding cinaciguat, as demonstrated by the heat map and volcano plot (Figures 5(a) and 5(b)). GO enrichment analysis showed the biological processes, molecular functions, and cellular components associated with DEPs, and a total of 25 KEGG pathway terms were enriched with statistical significance (Figure S1 A-D).

Furthermore, PPI analysis was constructed by STRING platform, and the network was visualized in cytoscape, which provided a 22-node protein module with PKG2 as the central hub protein, and 5 of those (Prkg1, Atp2b4, Pln, Vasp, and Plc*β*1) had a stronger relationship than the others, which connected with thicker lines in Figure 5(c). Based on comprehensive literature retrieval of the node proteins, we were able to recognize a putative regulatory relationship between PKG2 and PLC*β*1, whose expression was greatly increased in the T2DM medium. Specifically, PLC*β*1 activity is inhibited by PKG2 via phosphorylation of regulator of G protein signaling 4 (RGS4) [23]. Further, the activation of PLC*β*1 could lead to the hydrolysis of PIP2, generating two second messengers: diacylglycerol (DAG) and IP3 [24]. The latter could bind of IP3 receptor (IP3R), which is the Ca^2+^-release channel on the endoplasmic reticulum. When exposure to stress, Ca^2+^ excessive release through the IP3R results in the exhaustion of Ca^2+^ in the endoplasmic reticulum and the accumulation of Ca^2+^ in the cytoplasm [25], which disorders Ca^2+^ homeostasis triggering ER stress and accelerating cell damage. Based on the above, we suspected that upregulated PKG2 by cinaciguat could inhibit the activation of PLC*β*1, relieve intracellular calcium overload, and then suppress ER stress to ameliorate osteoblast functions under T2DM condition.

### 3.6. Cinaciguat Inhibited the Activation of PLC*β*1/IP3R via PKG2 under T2DM Condition

To verify the protein mass spectrometry results, the protein expression level of PLC*β*1 was detected by western blotting (Figure 5(d)). As expected, T2DM treatment led to higher expression of PLC*β*1 compared to the control group, while cinaciguat decreased the expression of PLC*β*1, which was consistent with proteomic analysis. Consequently, cinaciguat reduced the expression of IP3R compared with the T2DM group. The drug was impotent in *prkg2*-deficient cells, which represented the reducing effect was mediated by PKG2.

### 3.7. Cinaciguat Relieved Intracellular Calcium Overload and ER Stress via PKG2 by Suppressing PLC*β*1

**Figure 6:**
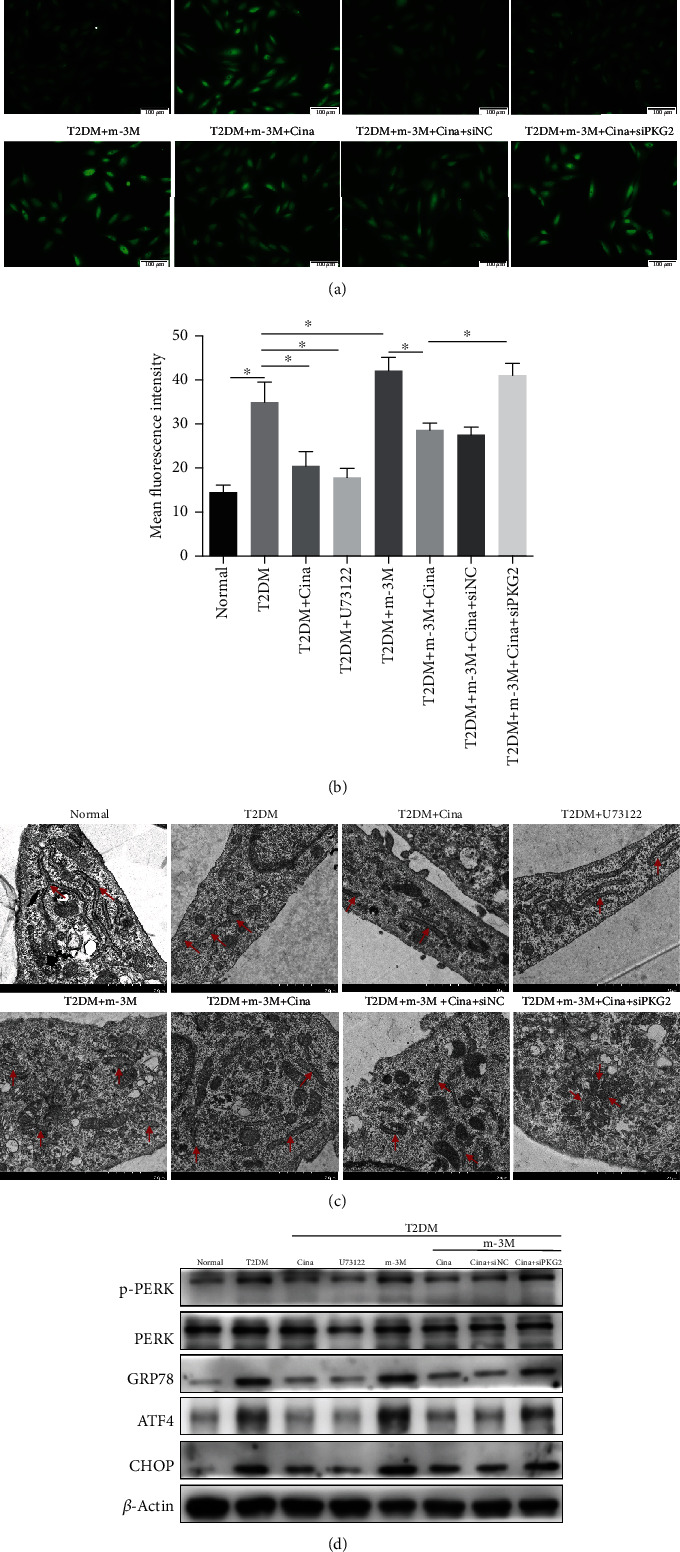
Cinaciguat relieved intracellular calcium overload and ER stress via PKG2 by suppressing PLC*β*1. (a) Images of intracellular Ca^2+^ fluorescence stained with Calbryte™ 520 AM. Scale bars = 100 *μ*m. (b) Quantitative analysis of mean fluorescence intensity of intracellular Ca^2+^. (c) Transmission electron microscopy images showed ER morphology after 3 days stimulation. ER is denoted by red arrows. Scale bars = 2 *μ*m. (d) The protein levels of p-PERK, GRP78, ATF4, and CHOP were detected by western blotting. Data were presented as means ± SD from at least three independent experiments. ∗: *p* < 0.05. Cina: cinaciguat; m-3M: m-3M3FBS.

To further understand the interaction between PKG2 and PLC*β*1, m-3M3FBS and U73122 were used to activate and inhibit PLC*β*1, respectively. The relevant effect of two drugs was verified by western blot (Figure S2). Meanwhile, the concentration of intracellular Ca^2+^was detected in osteoblast by Calbryte™ 520 AM, and the fluorescence intensity (Figures 6(a) and 6(b)) indicated that both cinaciguat and U73122 treatment could inhibit the accumulation of Ca^2+^ in the cytoplasm induced by T2DM medium. The addition of m-3M3FBS achieved more cytoplasm Ca^2+^ than the T2DM group, demonstrating that the activation of PLC*β*1 by m-3M3FBS aggravated calcium overload, while cinaciguat could mitigate this adverse effect. Also, cinaciguat was impotent to rescue the above adverse effect when knockdown of PKG2. Mounting evidences have shown that intracellular calcium increase could induce ER stress [26, 27]. In our study, ER stress response was assessed by TEM investigations (Figure 6(c)) and detecting the protein expression of ER stress markers (Figure 6(d)). T2DM stimulation increased the expression of phosphorylated PERK, GRP78, ATF4, and CHOP, while cinaciguat and U73122 inhibited the activation of the above proteins. To verify whether cinaciguat could restrain the activation of the ER stress through PLC*β*1, we added m-3M3FBS into osteoblasts. Western blot showed that p-PERK, GRP78, ATF4, and CHOP expression was higher in T2DM + m-3M3FBS group than in T2DM, which was reversed by exposing to cinaciguat and in siNC group. Additionally, the alterations of subcellular ER structure were detected by TEM. Gross ER dilation and distortions were observed in cells in response to T2DM, and the dilation was exacerbated after the addition of m-3M3FBS. Fortunately, the negative phenomena were largely repressed by U73122 treatment and reactivation of PKG2 by cinaciguat.

### 3.8. Cinaciguat Showed Osteoprotective Effects via PKG2 by Suppressing ER Stress under T2DM Condition

**Figure 7:**
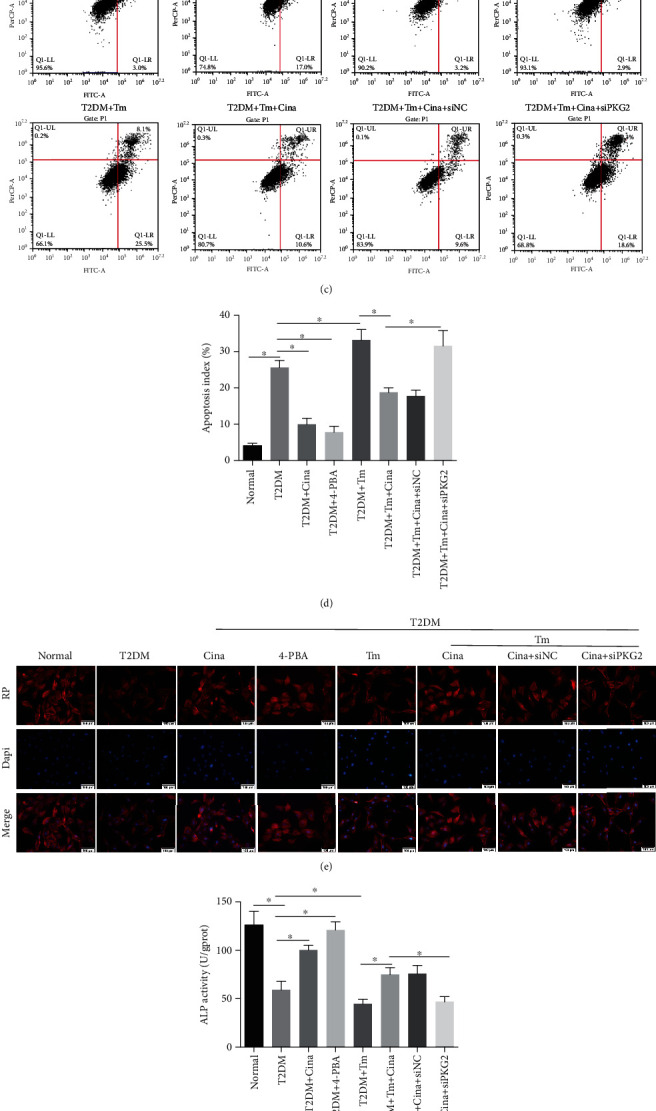
Cinaciguat showed osteoprotective effects via PKG2 by suppressing ER stress under T2DM condition. (a) EDU staining was performed to test the osteoblasts undergoing DNA replication. Red, EDU positive nucleus. Blue, all nucleus. Scale bar = 100 *μ*m. (b) The quantification analysis of EDU-positive cells. (c) Flow cytometer analysis was conducted to detect cell apoptosis. (d) The quantification analysis of apoptosis rate. (e) Cytoskeleton (red) was stained by rhodamine-phalloidin, and nucleus (blue) was stained by DAPI. Scale bars = 100 *μ*m. (f) ALP activity assay after 7 days stimulation. (g) ALP staining after 7 days stimulation. (h) The protein levels of COL1, ALP, and RUNX2 were detected by western blot after 7 days stimulation. Data were presented as means ± SD from at least three independent experiments. ∗: *p* < 0.05. RP: rhodamine-phalloidin; Cina: cinaciguat; Tm: tunicamycin; 4-PBA: 4-phenylbutyric aci.

To detect whether ER stress contributed to T2DM-related dysfunction in osteoblasts, we analyzed the impact of 4-phenylbutyric acid (4-PBA), an inhibitor of ER stress, and tunicamycin, an activator of ER stress, on the proliferation, apoptosis, and differentiation of cells. Western blot displayed that 4-PBA and tunicamycin could inhibit and activate the pathway of ER stress (Figure S3). Both cinaciguat and 4-PBA ameliorated DM-induced decreased proliferation (Figures 7(a) and 7(b)), excessive apoptosis (Figures 7(c) and 7(d)), disordered adhesion (Figure 7(e)), and impaired osteogenesis (Figures 7(f)–7(h)). Moreover, tunicamycin treatment alone under T2DM condition could aggravate these dysfunctions in osteoblasts. In addition, the inhibitory effects of tunicamycin on proliferation and differentiation were countered by the addition of cinaciguat, while the effects were disappeared in the siPKG2 group.

## 4. Discussion

Type 2 diabetes mellitus is closely related to impaired implant osteointegration, and increased risk of implantation failure, which is due to the function of osteoblasts, is suppressed under T2DM condition. So, it is very necessary to choose an ideal T2DM-simulated vitro model to explore the relationship between characteristics of osteoblasts and pathological microenvironment. There are many methods of imitating DM conditions in vitro [28], and the most used models are the addition of high glucose (HG) in culturing conditions. However, the effects of HG alone on osteoblasts have led to a controversy, with either inconsistent or contradictory results. Some researchers revealed that hyperglycemia decreased proliferation, differentiation of osteoblasts [29, 30]. In contrast, a few researchers indicated that hyperglycemia promoted mineralization and increased the expression of osteogenesis-related genes in osteoblasts [31]. Compared to type 1 diabetes mellitus (T1DM), T2DM is a more complicated metabolic disease, characterized by not only hyperglycemia but also dyslipidemia [1, 32]. Therefore, we tried to clarify the influence of both high glucose (D-glucose, 25 mM) and high lipid (palmitate, 200 *μ*M) on osteoblasts, helping to more closely imitate diabetes condition and subsequent impaired implant osteointegration. In this study, we first detected essential osteogenesis characteristics of osteoblasts on Ti using T2DM-simulated vitro model, as evidenced by decreased EDU-positive cells as well as increased cell apoptosis, together with anomalous cell adhesion and poor cell differentiation. All of the pathological changes were alleviative in the normal and control groups. These results offered evidence that diabetes-induced dysfunction of osteoblasts on Ti surface was established with success.

PKG2 is the main target of cGMP in osteoblasts and osteocytes [33], whose deficiency could cause skeletal abnormalities in humans [34, 35]. Further, we have previously confirmed that the poor implant osseointegration observed in T2DM rats is partly caused by impaired cGMP/PKG2 pathway in osteoblasts, while the precise mechanism is still unclear. In this study, we focused on the expression of PKG2 in vitro and discovered that it was downregulated in diabetic-stimulated osteoblasts, which was proved by western blot and immunofluorescence. Similar to phenomenon appeared in osteoblasts, several studies have indicated the reduced PKG expression in cardiomyocytes [15, 36] and cytotrophoblast cells [37] under diabetic conditions. In order to determine if promoting PKG2 expression is sufficient to protect osteoblasts from diabetes-induced dysfunction, we increased its activity by transfecting lentivirus and adding cinaciguat. The overexpression of PKG2 by lentivirus promoted cell proliferation, adhesion, and differentiation, leading to decreased osteoblasts injury. The same protective effect also happened to the cinaciguat group. We started by measuring the recovery of PKG2 expression, producing about two-fold increased PKG2 protein in the T2DM + Cina group versus the T2DM group, which demonstrated that PKG2 activity was elevated in a close to normal physiological range. As expected, the PKG2 gain of function gave rise to cell-increased in proliferation, cell-inerratic in adhesion, and cell-resistive in apoptosis, explaining ameliorative differentiation ability of osteoblasts. Also, we further afforded evidence that cinaciguat improved diabetic-induced dysfunction of osteoblasts mediated by PKG2, because when PKG2 was silenced, the opposite results were observed.

The above results and our previous study demonstrated that cinaciguat could attenuate T2DM-induced osteoblast injury and poor osseointegration. However, the precise mechanisms leading to the protective effects of cinaciguat remain to be elucidated. In this study, we conducted proteomic analysis to enable the detection of some low abundance proteins, offering a more accurate picture of the pathways regulated by cinaciguat under T2DM condition. This deep proteomic coverage then identified a link between the downregulation of PLC*β*1 and upregulation of PKG2 in adding the cinaciguat group. PLC*β*1 is the major PLC isoform located on the nuclear envelope [38]. Data of the proteomics and western blot displayed that PLC*β*1 was highly expressed in the T2DM group and inhibited in T2DM + cina. Moreover, the inhibitory effect was mediated by PKG2, which is consistent with PPI analysis. Current evidence suggests that excessive activation of PLC*β*1 under diabetic conditions means to generate much more IP3, so as to activate IP3R [39], the Ca^2+^ channel on the ER. Wozniak et al. indicated that PLC*β*1 could encourage Ca^2+^ release through IP3R from the ER, resulting in a high cytoplasmic Ca^2+^ concentration [40]. Although Ca^2+^ is a crucial second messenger that regulates many processes of cellular biology, the excessive accumulation of intracellular Ca^2+^ could trigger ER stress, which eventually causes damage to cells and leads to many diseases [27, 41]. Thus, we hypothesized that ER stress may be involved in diabetic injury, inducing osteoblast dysfunction and poor osseointegration, and cinaciguat could ameliorate the adverse effects by regulating ER stress via the PLC*β*1 inhibition. To verify the hypothesis, we exposed osteoblasts to cinaciguat, U73122 (PLC inhibitor), and m-3M3FBS (PLC activator) under T2DM condition. Calcium fluorescence was used to evaluate the concentrations of Ca^2+^ in cytoplasm, and the result indicated that both cinaciguat and U73122 could decrease the cytoplasmic Ca^2+^ induced by diabetic condition. When PLC*β*1 activated by m-3M3FBS, there was a higher cytoplasmic Ca^2+^ than the T2DM group and then was suppressed by cinaciguat. Moreover, the depressor effect of cinaciguat had gone off while PKG2 silencing. Normally, chaperone GRP78 binds to ER stress sensor, PERK, and inhibits its activation [42]. When undesirable stimulation or damage triggers calcium overload, GRP78 dissociates from PERK and subsequently leads to ER stress. The process promotes the translation of ATF4 and activation of CHOP [43]. Our results indicated that T2DM treatment remarkably activated the ER stress-related proteins of osteoblast, which agreed with previous findings that palmitic acid could induce the high expression of GRP78 and CHOP in osteoblast-like Saos-2 cell [44]. Furthermore, we discovered that m-3M3FBS-induced the worse ER stress in osteoblasts was suppressed by cinaciguat mediated by PKG2. These observations suggested that diabetes generates ER stress in osteoblasts that is dependent on Ca^2+^ homeostasis; moreover, cinaciguat relieved intracellular calcium overload and ER stress via PKG2 by suppressing PLC*β*1, providing a novel pharmaceutical target for cinaciguat.

Then, we explored the interaction between osteoblast dysfunction and ER stress. Tunicamycin, a N-linked glycosylation inhibitor, could induce ER stress, and 4-PBA could promote protein folding and secretion to suppress ER stress. Both of them were added in medium to examine the anti-ER stress effects of cinaciguat. Our data showed that 4-PBA could restrain T2DM-induced decrease in proliferation, disorder adhesion, and poor differentiation in osteoblasts, especially in excessive apoptosis. Similarly, Guan et al. also provided that palmitic acid could activate ER stress to induce apoptosis in cardiomyocytes, and 4-PBA could reduce apoptosis by inhibition of ER stress [45], which further expanded our results that T2DM induced osteoblast dysfunction via ER stress. The similar effects were achieved with the application of cinaciguat. With the addition of tunicamycin, osteoblasts suffered the most damage in all groups, and the adverse effect of tunicamycin was inhibited by cinaciguat. However, there was more serious osteoblast injuries in the siPKG2 group than in the siNC group, which indicate that cinaciguat played anti-ER stress role through PKG2. The above findings showed that the excessive activation of ER stress was involved in osteoblast dysfunction under diabetic environment, and cinaciguat played an osteoprotective role via suppressing the ER stress activation.

## 5. Conclusions

**Figure 8:**
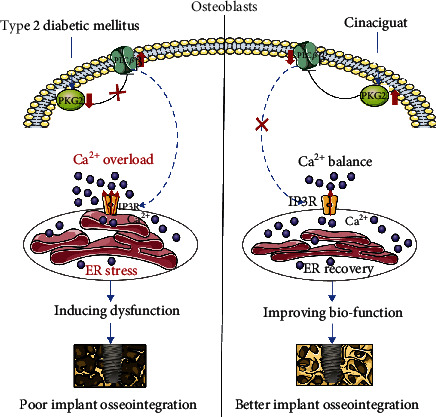
Schematic illustration of the detailed mechanisms responsible for cinaciguat provided a favorable effect on promoting osseointegration in T2DM.

In summary, our T2DM-simulated vitro model could impair the biological characters of osteoblasts on Ti disc, and cinaciguat could alleviate this injury by reactivation PKG2. Moreover, integrated proteomic analysis identified a link between PKG2 and PLC*β*1 in adding cinaciguat group. Then, we basically demonstrated that cinaciguat inhibited the PLC*β*1 activation to attenuate the intracellular Ca^2+^ overload-ER stress pathway and protected the osteoblasts from diabetes. These results provide the first detailed mechanisms responsible for cinaciguat treatment provided a favorable effect on promoting osseointegration in T2DM (Figure 8). Furthermore, the present study provides a new insight that diabetes mellitus-induced the aberrations in PKG2-PLC*β*1-Ca^2+^-ER stress pathway is one underlying mechanism for poor osseointegration, which might be key therapeutic targets to promote osteointegration in T2DM patients.

## Data Availability

The data used to support the findings of this study are available from the corresponding author upon request.

## Supplementary Materials

Supplementary Materials Figure S1: (a) enrichment analysis of KEGG pathways for the differentially expressed proteins. Enrichment analysis of GO terms for the differentially expressed proteins, including (b) cellular component, (c) molecular function, and (d) biological process categories. Figure S2: the protein level of PLC*β*1 was detected by western blotting to verify the relevant effect of U73122 and m-3M3FBS. m-3M: m-3M3FBS. Figure S3: the protein levels of p-PERK, GRP78, ATF4, and CHOP were detected by western blotting to verify the relevant effect of 4-PBA and tunicamycin. 4-PBA: 4-phenylbutyric acid; Tm: tunicamycin.
