# Supplementary material for: Pharmic Activation of PKG2 Alleviates Diabetes-Induced Osteoblast Dysfunction by Suppressing PLCβ1-Ca2+-Mediated Endoplasmic Reticulum Stress
Source: Oxid Med Cell Longev. 2021 Jun 16;2021:5552530. doi: 10.1155/2021/5552530 (PMC8225424; doi:10.1155/2021/5552530)
Supplement: Supplementary Materials — Figure S1: (a) enrichment analysis of KEGG pathways for the differentially expressed proteins. Enrichment analysis of GO terms for the differentially expressed proteins, including (b) cellular component, (c) molecular function, and (d) biological process categories. Figure S2: the protein level of PLCβ1 was detected by western blotting to verify the relevant effect of U73122 and m-3M3FBS. m-3M: m-3M3FBS. Figure S3: the protein levels of p-PERK, GRP78, ATF4, and CHOP were detected by western blotting to verify the relevant effect of 4-PBA and tunicamycin. 4-PBA: 4-phenylbutyric acid; Tm: tunicamycin. [file 5552530.f1.zip › Supplementary Materials/Supplementary materials (1).docx]

**Supplementary materials
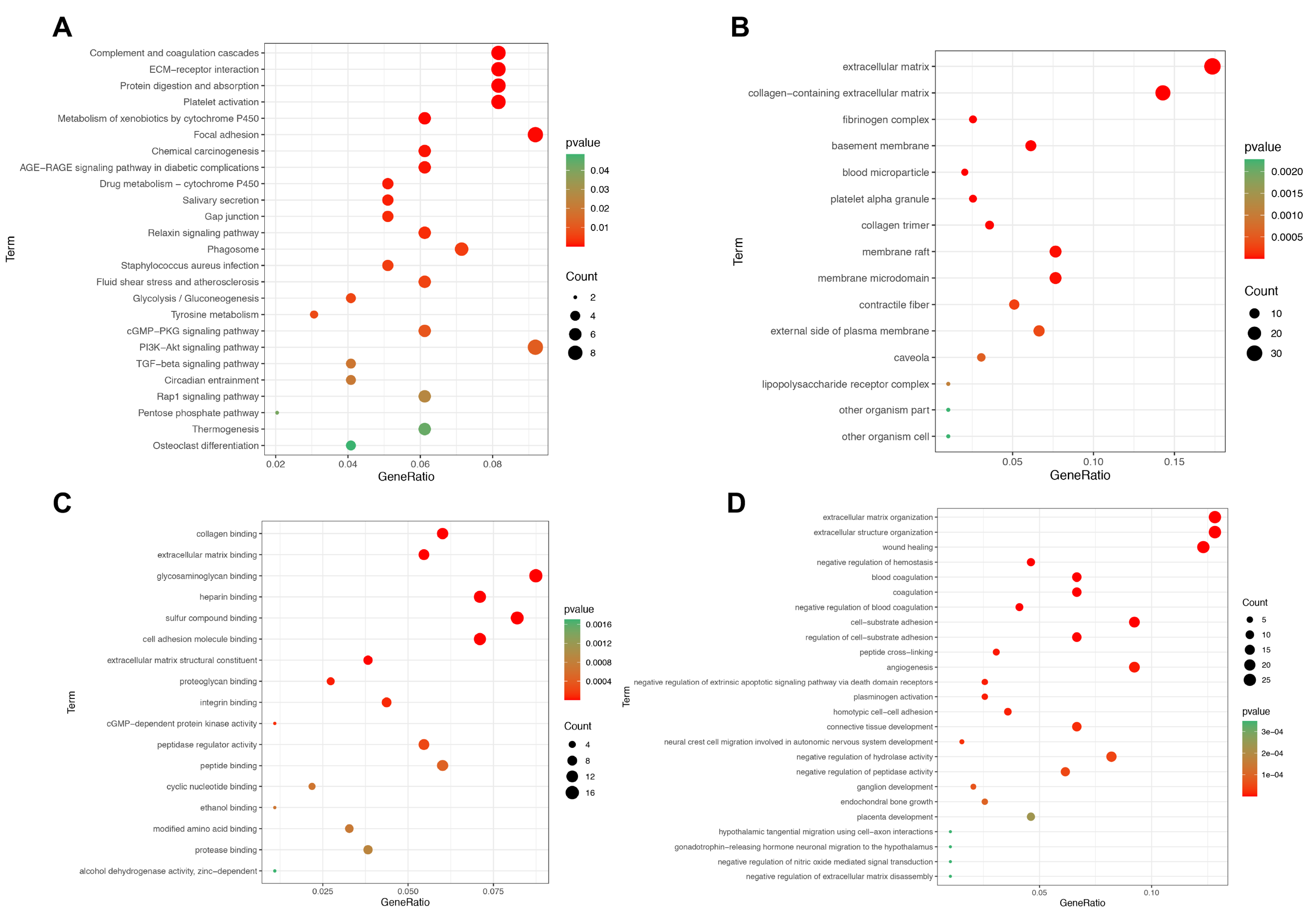
**

**Figure. S1.** (A) Enrichment analysis of KEGG pathways for the differentially expressed proteins. Enrichment analysis of GO terms for the differentially expressed proteins, including (B) cellular component, (C) molecular function and (D) biological process categories.


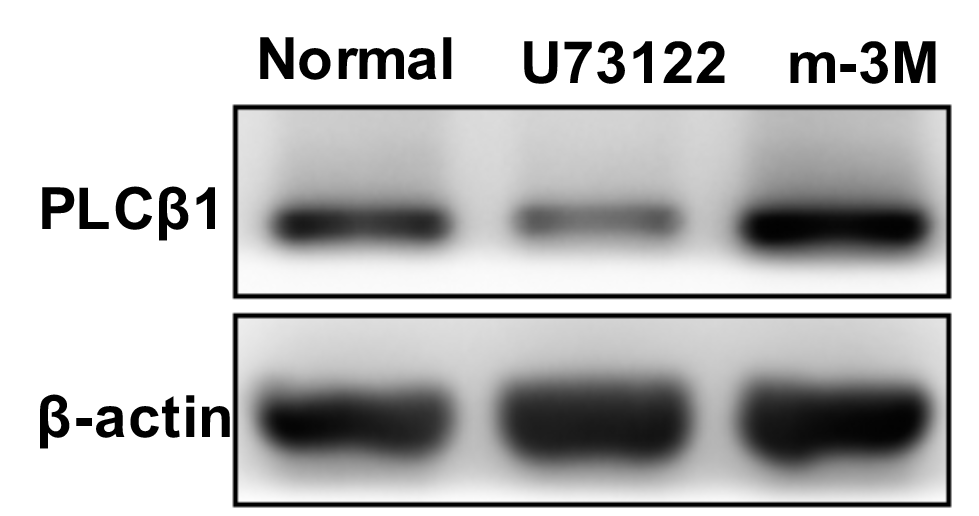


**Figure. S2.** The protein level of PLCβ1 was detected by western blotting to verify the relevant effect of U73122 and m-3M3FBS. m-3M: m-3M3FBS.


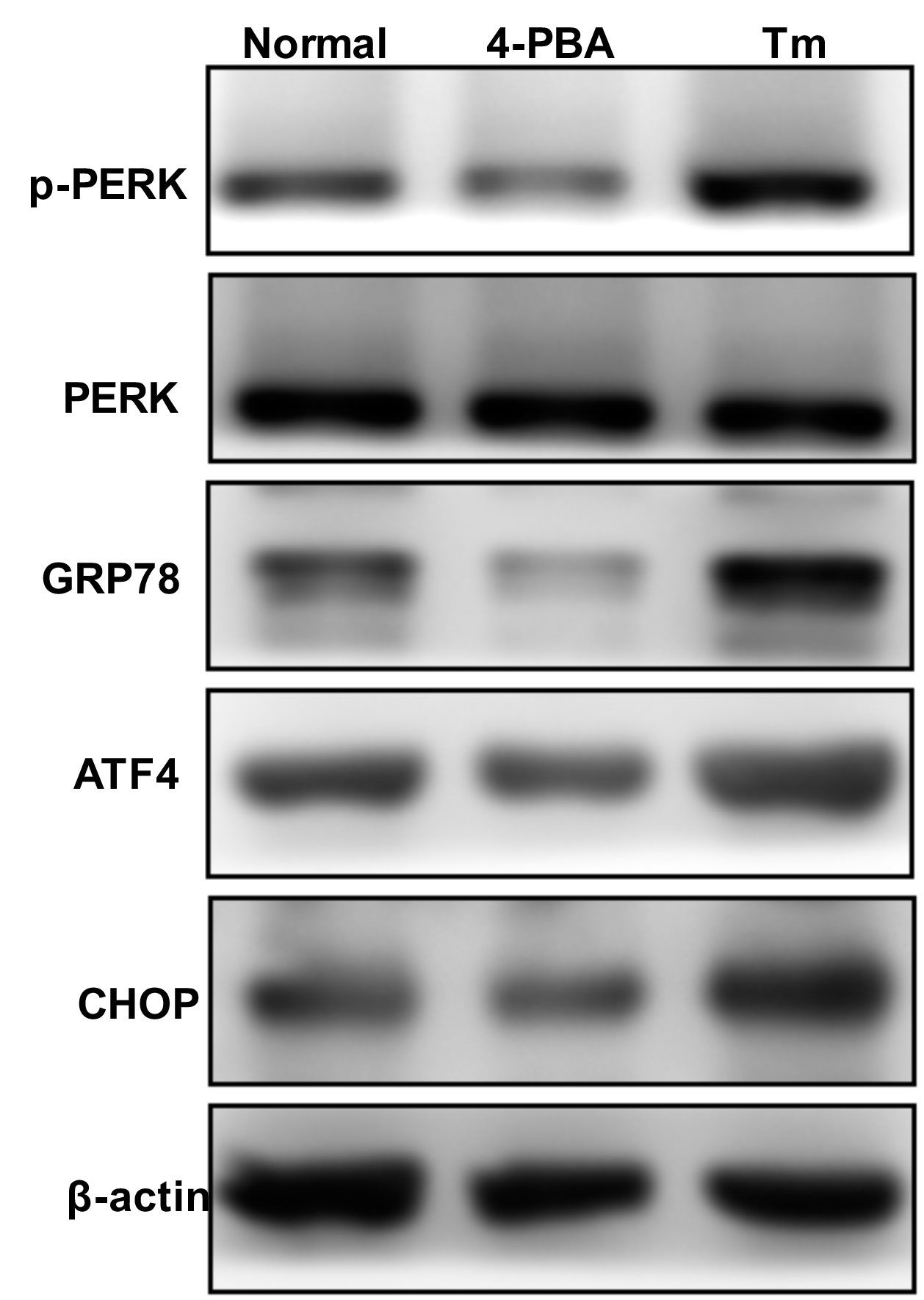


**Figure. S3.** The protein levels of p-PERK, GRP78, ATF4 and CHOP were detected by western blotting to verify the relevant effect of 4-PBA and tunicamycin. 4-PBA:4-phenylbutyric acid. Tm: tunicamycin.
